# Effects of Sucralose Ingestion versus Sucralose Taste on Metabolic Responses to an Oral Glucose Tolerance Test in Participants with Normal Weight and Obesity: A Randomized Crossover Trial

**DOI:** 10.3390/nu12010029

**Published:** 2019-12-20

**Authors:** Alexander D. Nichol, Clara Salame, Kristina I. Rother, M. Yanina Pepino

**Affiliations:** 1 Department of Food Science and Human Nutrition, University of Illinois at Urbana-Champaign, Urbana, IL 61822, USA; adnicho2@illinois.edu; 2 Division of Nutritional Sciences, University of Illinois at Urbana-Champaign, Urbana, IL 61822, USA; csalame2@illinois.edu; 3 National Institutes of Health, Bethesda, MD 20892, USA; kristina.rother@nih.gov

**Keywords:** low-calorie sweeteners, artificial sweeteners, non-nutritive sweeteners, sucralose, glucose metabolism, oral glucose tolerance test, insulin

## Abstract

Here, we tested the hypothesis that sucralose differentially affects metabolic responses to labeled oral glucose tolerance tests (OGTTs) in participants with normal weight and obesity. Participants (10 with normal weight and 11 with obesity) without diabetes underwent three dual-tracer OGTTs preceded, in a randomized order, by consuming sucralose or water, or by tasting and expectorating sucralose (e.g., sham-fed; sweetness control). Indices of β-cell function and insulin sensitivity (S_I_) were estimated using oral minimal models of glucose, insulin, and C-peptide kinetics. Compared with water, sucralose ingested (but not sham-fed) resulted in a 30 ± 10% increased glucose area under the curve in both weight groups. In contrast, the insulin response to sucralose ingestion differed depending on the presence of obesity: decreased within 20–40 min of the OGTT in normal-weight participants but increased within 90–120 min in participants with obesity. Sham-fed sucralose similarly decreased insulin concentrations within 60 min of the OGTT in both weight groups. Sucralose ingested (but not sham-fed) increased S_I_ in normal-weight participants by 52 ± 20% but did not affect S_I_ in participants with obesity. Sucralose did not affect glucose rates of appearance or β-cell function in either weight group. Our data underscore a physiological role for taste perception in postprandial glucose responses, suggesting sweeteners should be consumed in moderation.

## 1. Introduction

A high-calorie diet with excess sugar intake increases the risk of developing obesity, cardiovascular disease, and type 2 diabetes [1]. Therefore, limiting the consumption of added sugar is recommended by many organizations around the world [2]. Because low-calorie sweeteners (LCSs) provide intense sweet taste with few if any calories, the use of LCSs as sugar substitutes is becoming increasingly popular; it is estimated that about 25% of children and 41% of adults in the United States consume LCSs [3].

Even though LCSs have a negligible calorie content, data from animal models and human cell lines suggest that they are metabolically active, presumably by interaction with sweet taste receptors [4,5,6,7,8,9,10,11,12]. The sweet taste receptor T1R2 + T1R3 is a heterodimer that detects sugars and LCSs and is expressed in many tissues beyond the mouth, including the intestine and the pancreas [4]. Activation of these sweet taste receptors in the intestine results in a faster rate of glucose absorption [5,6,7,8,13] and in the secretion of incretins [9], although data from studies in vivo suggest that the extent of incretin secretion varies with different sweet taste ligands [14,15]. In addition, activation of these receptors in the pancreas can result in increased insulin secretion [10,11,12] and in the mouth, it can elicit pre-ingestive cephalic-phase responses, including a cephalic-phase insulin response (CPIR), which are thought to prime the body to better absorb and use ingested nutrients [16,17].

We recently found that the ingestion of sucralose before an oral glucose tolerance test (OGTT) increased the plasma insulin response and decreased whole-body insulin sensitivity in people with obesity [18]. However, it is unknown whether this effect was mediated by the perception of sucralose sweet taste in the mouth or by sucralose post-oral effects. Further, data from most studies conducted in people who are normal weight show that acute sucralose ingestion does not affect the plasma glucose or insulin response to carbohydrate ingestion [15,19,20,21]. These findings suggest that acute sucralose ingestion has different effects in people who are obese than in those who are normal weight.

The primary goals of this study were to distinguish the acute effects of sucralose ingestion from the perception of the sweet taste of sucralose per se on the metabolic response to glucose ingestion, and to compare such acute effects of sucralose in people who are normal weight and those who are obese. We hypothesized that, compared with the ingestion of water before a glucose load, the ingestion of sucralose before a glucose load, but not sucralose taste alone (i.e., sucralose tasted and expectorated, hereafter sham-fed), would cause a greater increase in glucose-stimulated insulin response (as we found previously [18]) and impair the suppression of endogenous glucose production (EGP) in participants with obesity but not in those who are normal weight. A secondary goal of the study was to assess plasma sucralose concentrations achieved after sucralose ingestion. We hypothesized that people with obesity would be more vulnerable to the adverse metabolic effects of sucralose ingestion than those with a normal weight, in part because the increased intestinal permeability associated with obesity [22] would enhance the absorption of ingested sucralose and increase plasma sucralose concentrations.

## 2. Materials and Methods

### 2.1. Study Overview

Using a randomized crossover design, participants were evaluated on three separate study visits. We chose this type of study design, in which each subject acts as his or her own control, to reduce the potential influence of confounding factors, such as dietary variations and baseline glucose metabolic characteristics. The study visits took place at the same times, approximately one week apart. For each study visit, participants were admitted at ~07:00 to the Clinical Research Unit at Washington University School of Medicine. Participants were instructed to fast overnight (12 h) at home and to avoid physical exercise for 3 days before each study visit. After vital signs were obtained, one catheter was inserted into a forearm vein for infusion, and a second catheter was inserted into a radial artery to obtain blood samples. A primed, continuous infusion of (6,6-2H2) glucose (priming dose, 22 µmol/kg; infusion rate of 0.22 µmol/kg·min) was started and maintained until the end of the study. After 3.5 h of tracer infusion, participants ingested a drink containing 75 g glucose mixed with 1.5 g [U-13C6] glucose. Blood samples were obtained at 40, 30, 20, 10, 8, 6, 4, and 2 min before and at 0, 10, 20, 30, 40, 50, and 60 min after ingestion of glucose, and then every 20 min for an additional 4 h to determine glucose kinetics, and plasma hormone and sucralose concentrations. In a randomized order, 10 min prior to glucose ingestion, participants drank 60 mL of 2 mmol/L sucralose (i.e., 48 mg sucralose, as in our previous study [18]) or an equivalent volume of distilled water, or they sham-fed sucralose. The sham-feeding procedure consisted of swishing the same amount of sucralose in their mouth for ~5 s and then spitting it out.

### 2.2. Participants

Participants were enrolled in the study between April 2015 and August 2016. They completed a screening visit that included a standard 75 g 2 h OGTT and a questionnaire used in previous studies that inquired about typical LCSs use [18,23]. Participants with a plasma glucose concentration ≥7 mmol/L at fasting or ≥11.1 mmol/L at 2 h post-OGTT, or with significant insulin resistance (based on a homeostatic model assessment of insulin resistance score (HOMA-IR2)) > three were excluded from participation. Also, those who were regular consumers of LCSs (i.e., reported consuming more than one diet beverage per week, one spoonful of LCSs per week, or an equivalent amount of LCSs in foods); smoked tobacco cigarettes in the past six months; were pregnant or breastfeeding; had a history of malabsorptive syndromes, bariatric surgery, or inflammatory intestinal disease; or were taking any medication that may affect glucose metabolism were excluded. The study conformed to the revised Declaration of Helsinki and was approved by the institutional review board at Washington University School of Medicine in St. Louis, MO, USA. All screened participants gave informed written consent before participation. The study was registered at ClinicalTrials.gov, with the identifier: NCT02413424.

### 2.3. Laboratory Assessments

At all time points, plasma glucose was measured immediately after collection using an automated glucose analyzer (YSI 2300 STAT Plus; Yellow Springs Instruments, Yellow Springs, OH, USA). Blood samples were also collected in chilled EDTA tubes containing a protease inhibitor cocktail (Millipore, Billerica, MA, USA). These samples were placed on ice and centrifuged at 4 °C, and the plasma was stored at −80 °C for subsequent analyses. Plasma insulin and C-peptide concentrations were measured at the same time points as plasma glucose up to 60 min post-glucose ingestion and then every 40 min until the end of the test. Plasma insulin concentrations were determined using a two-site immunoenzymatic assay (DxI 800; Beckman Instruments, Chaska, MN, USA), and C-peptide by using a solid-phase two-site chemiluminescent immunometric assay (Siemens Medical Solutions Diagnostics, Los Angeles, CA, USA). Plasma glucose-dependent insulinotropic peptide (GIP) was measured at 10 and 2 min before and at 0, 10, 30, 50, 60, 100, 140, and 180 min after glucose consumption using commercially available immunoassay kits from Millipore. The glucose tracer-to-tracee ratio in plasma was determined using GC-MS (Hewlett-Packard MSD 5973 system with a capillary column) after derivatization with acetic anhydride. Plasma sucralose concentrations were measured 10 min prior, immediately before, and at 10, 40, 70, 130, 190, and 310 min after sucralose consumption using liquid chromatography-mass spectrometry, as described previously [24].

### 2.4. Calculations

Metabolic response to glucose ingestion. The above baseline concentrations—incremental areas under the curve (iAUC) for glucose, insulin, C-peptide, and GIP—were calculated using the trapezoidal method [25].

#### 2.4.1. Insulin Sensitivity

We used the oral glucose minimal model as described previously [26] to estimate insulin sensitivity (S_I_; dL·kg^−1^·min^−1^/(pmol/L)).

#### 2.4.2. β-Cell Function and Insulin Clearance

We used the oral C-peptide minimal model as described previously [27] to estimate the insulin secretion rate (ISR) in response to the oral glucose load and the sensitivity of the β-cell response to changes in plasma glucose. This model provides an estimate of the total amount of insulin secreted in response to plasma glucose as a function of time (i.e., total ISR, in pmol/min). This model also partitions the total ISR response into a dynamic component (ISRdynamic), representing the rapid release of a “readily releasable pool” of insulin secretory granules in response to the rate of increasing plasma glucose concentration, and a static component (ISRstatic), representing the slower release of a “reserve pool” of insulin secretory granules in response to the ambient plasma glucose concentration [27]. We also determined the β-cell response sensitivity parameters (Φtotal, Φdynamic, Φstatic) corresponding to the total, dynamic, and static ISR in response to changes in plasma glucose. We estimated the insulin clearance rate (pools/min) from plasma by dividing the iAUC of ISR by the iAUC of plasma insulin concentration. We then multiplied the insulin clearance rate by the volume of distribution for C-peptide from the ISR modeling (used as the volume of distribution for insulin) to express insulin clearance rate as L/min.

#### 2.4.3. Glucose Kinetics

Total glucose rate of appearance (Total Ra) into systemic circulation during the OGTT comes from both ingested glucose and EGP. Total glucose rate of appearance and EGP were determined as previously reported ([28] and Supplementary Methods in Appendix A). The oral glucose rate of appearance (Oral Ra) was calculated as the difference between Total Ra and EGP [28,29].

### 2.5. Statistical Analyses

To determine the statistical significance of the effect of tasting or tasting and ingesting sucralose on plasma glucose, insulin, C-peptide, and GIP concentrations, as well as glucose kinetics (Total Ra, oral glucose rate of appearance, and EGP), ISR, S_I_, and insulin clearance during the OGTT, and to determine the effect of obesity on plasma sucralose concentrations, we used the general linear mixed model (PROC MIXED) analysis with subject-level random intercepts. Treatment (water, sucralose, and sham-fed), time (when applicable), and weight group (normal weight and obese), as well as all interactions (when applicable) were included in the model and treated as fixed effects. When differences in values were statistically significant, post hoc Fisher least significant difference analyses were conducted. Because the PROC MIXED analysis can handle unbalanced designs, data from two participants in the group with obesity who did not complete the sucralose sham-fed visits were also included (therefore, for the group with obesity PROC MIXED analyses included data on 11 participants for sucralose and water treatment and 9 participants for sham-fed). Data in the tables and figures are presented as the mean ± SEM unless otherwise stated. All analyses were performed with SAS 9.3 (SAS Institute, Cary, NC, USA), and criterion for statistical significance was *p* ≤ 0.05.

The primary outcome measure of this study was the comparison of the differences on insulin responses to glucose on the sucralose treatment versus water treatment between weight groups. Based on data from our previous sucralose study [18], we estimated that 10 participants in each weight group would allow us to detect a large effect (0.9) on insulin response between groups with a β-value of 0.20 (i.e., 80% power) and a α-value of 0.05. For the power calculation, we used a multivariate linear model for repeated measures with PROC GLMPower procedure using the Hotelling-Lawley Trace statistics and LEAR correlation for the structure of the covariance (SAS 9.3).

## 3. Results

### 3.1. Participant Characteristics

**Figure 1:**
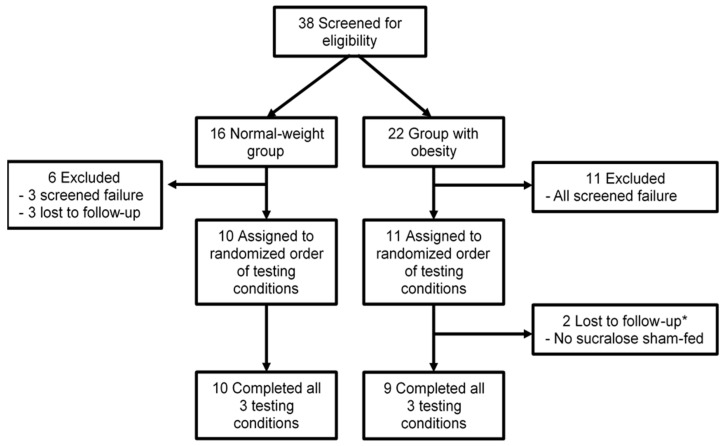
Study design flow diagram. * Because the PROC MIXED analysis can handle unbalanced designs, data from the two participants who did not complete one of the study visits were also included.

Thirty-eight potential participants (16 normal-weight participants and 22 participants with obesity) enrolled in the study. Of those 38, 14 failed screening, three were lost to follow up after their screening visit, and two did not complete one of the study visits (Figure 1). There were no significant differences in age, sex, or fasting plasma glucose concentrations between weight groups (Table 1). However, mean fasting plasma insulin concentrations and HOMA-IR2 were significantly higher in the group with obesity than in the normal-weight group (Table 1).

### 3.2. Plasma Glucose and Hormone Concentrations

**Figure 2:**
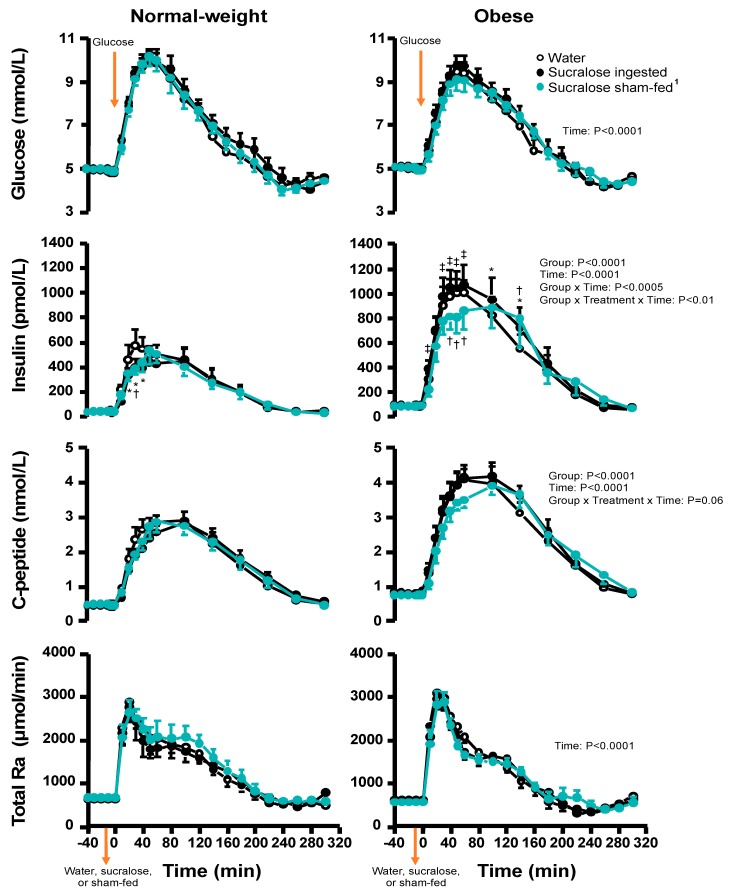
Plasma glucose, insulin, C-peptide concentrations, and total glucose rate of appearance into systemic circulation in participants with normal weight (*n* = 10; left panels) and participants with obesity (*n* = 11; right panels) after ingesting either sucralose or water or tasting but not swallowing sucralose (sham-fed) 10 min before ingesting a drink containing 75 g glucose at time 0 min^. 1^ *n* = 9 participants for sucralose sham-fed condition in the obese group. The general linear mixed model (PROC MIXED) analysis, which can handle an unbalanced designed, was used. Treatment (water, sucralose, and sham-fed), time, and weight group (normal weight and obese), as well as all interactions were included in the model and treated as fixed effects and subject was included as random effect. Post hoc Fisher least significant difference was used when interactions were statistically significant. * Value from sucralose treatment significantly different from corresponding water treatment, *p* < 0.05; † Value from sham-fed treatment significantly different from corresponding water treatment, *p* < 0.05; ‡ Value from sucralose treatment significantly different from corresponding sham-fed treatment, *p* < 0.05. All values are the mean ± SEM.

**Figure A1:**
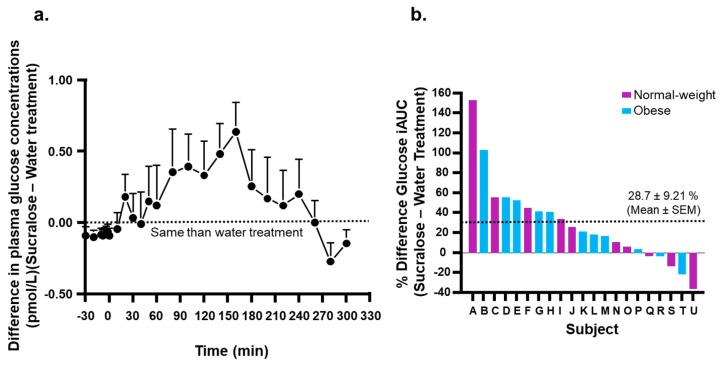
(**a**) Average difference in plasma glucose concentrations between water and sucralose treatment for all participants (normal-weight and obese groups combined). (**b**) Percent difference in the incremental area under the curve (iAUC) for glucose for each participant during the day they ingested sucralose compared with the day they ingested water 10 min before the glucose load in participants with normal weight (purple bars) and participants with obesity (blue bars).

**Figure A2:**
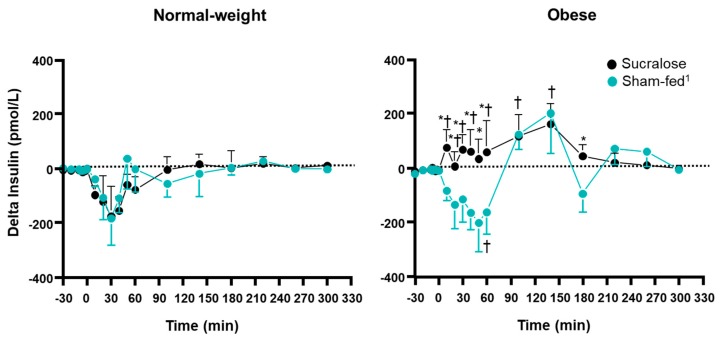
Changes in insulin concentrations relative to the water treatment at each given time point for the sucralose treatment and for the sham-fed treatment in the group of participants with normal weight (*n* = 10, left panel) and with obesity (*n* = 11, right panel). ^1^ *n* = 9 participants for sucralose sham-fed condition in the obese group. The dotted line represents values which are identical to those in water treatment. * Value from sham-fed treatment significantly different from water treatment within body-weight group at *p* < 0.05. ^†^ Value from same treatment significantly different between weight groups at *p* < 0.05.

**Figure A3:**
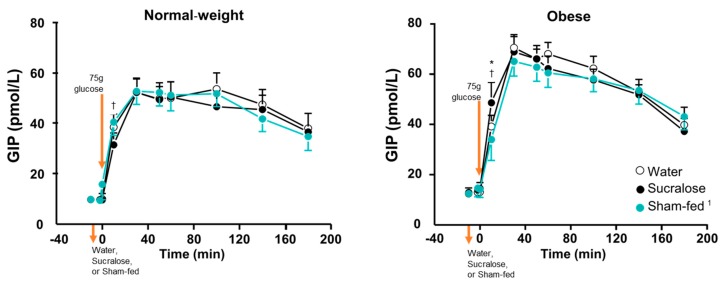
Plasma glucose-dependent insulinotropic peptide (GIP) mean concentration in normal-weight participants (*n* = 10; left panel) and participants with obesity (*n* = 11; right panel) after sucralose, water, or sham-fed treatment 10 min before ingestion of a 75 g glucose load at time 0 min. ^1^ *n* = 9 participants for sucralose sham-fed condition in the obese group. The general linear mixed model (PROC MIXED) analysis with subject-level random intercepts was used. Treatment (water, sucralose, and sham-fed), time, and weight group (normal-weight and obese), as well as all interactions, were included in the model and treated as fixed effects. Post hoc Fisher least significant difference was used when values were statistically significant. * Value from sucralose treatment significantly different from corresponding water treatment, *p* < 0.05; ^†^ Value from sucralose treatment significantly different from sham-fed treatment, *p* < 0.05. Data are presented as the mean ± SEM.

Sucralose ingested, but not sham-fed, increased the iAUC of plasma glucose by 30 ± 10% in both weight groups similarly (Treatment: F_(2,36)_ = 3.99; *p* = 0.03; Table 2, Figure A1 in Appendix B), and the peak response in plasma glucose concentrations tended to be lower in participants with obesity than in normal-weight participants (*p* = 0.06; Table 2). Plasma insulin and C-peptide mean and peak concentrations, as well as iAUCs, were significantly higher in participants with obesity than in normal-weight participants (all *p*-values < 0.02; Figure 2, Table 2). Compared with water treatment, sham-fed sucralose similarly reduced plasma insulin concentrations within the first hour of the OGTT in both weight groups, whereas sucralose ingestion differentially affected insulin concentrations in the two weight groups (Treatment × Weight Group × Time: F_(40,720)_ = 1.63; *p* = 0.009; Figure 1). While in normal-weight participants sucralose decreased plasma insulin concentrations during the first 40 min after the glucose load, in participants with obesity it resulted in significantly higher insulin concentrations compared with sham-fed sucralose (at 10 min, 30–60 min) and with water at 100–140 min after the glucose load (Figure 1; Figure A2 in Appendix B). The effects of sucralose, ingested and sham-fed, on C-peptide concentrations were similar to those described above for insulin, but the interaction between treatment, weight group, and time was not statistical significant (F_(40,720)_ = 1.37; *p* = 0.06; Figure 1). Plasma GIP mean and peak concentrations, as well as GIP iAUC, were not significantly different between weight groups (Figure A3 in Appendix B, Table 2). Compared with sham-fed sucralose, ingested sucralose affected plasma GIP concentrations 10 min post-OGTT in both weight groups, but in opposite ways (Treatment × Weight Group × Time: F_(18,323)_ = 2.55; *p* < 0.001): it decreased plasma GIP concentration in normal-weight participants but increased it in participants with obesity (Figure A3 in Appendix B). There were no significant differences among treatments or interactions for GIP iAUC (Table 2).

### 3.3. β-Cell Response

**Figure A4:**
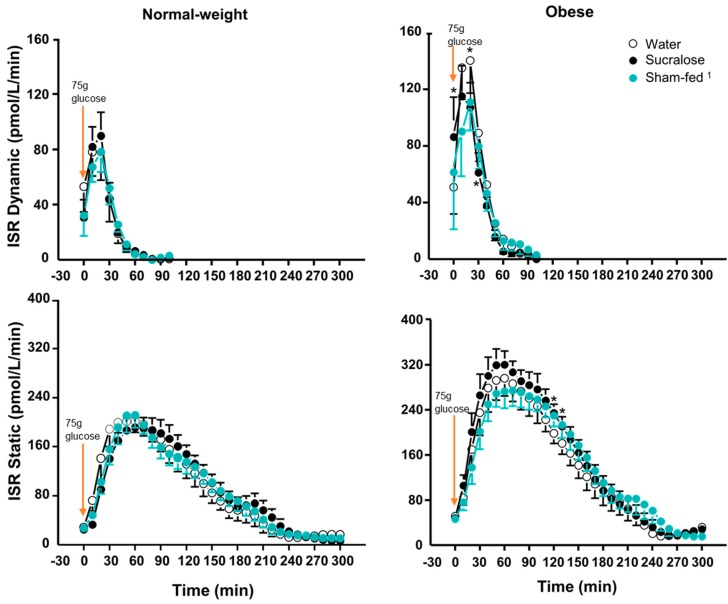
Insulin secretion rate (ISR) in response to a glucose load in normal-weight participants (*n* = 10; left panel) and participants with obesity (*n* = 11; right panel) after sucralose, water, or sham-fed treatment 10 min before ingestion of a 75 g glucose load at time 0 min. ^1^ *n* = 9 participants for sucralose sham-fed condition in the obese group. ISR is partitioned in its dynamic (top panels) and static (bottom panels) components. The general linear mixed model (PROC MIXED) analysis with subject-level random intercepts was used. Treatment (water, sucralose, and sham-fed), time, and weight group (normal-weight and obese), as well as all interactions, were included in the model and treated as fixed effects. Post hoc Fisher least significant difference was used when values were statistically significant. * Value from sucralose treatment significantly different from corresponding water treatment, *p* < 0.05. Data are presented as the mean ± SEM.

Total, dynamic, and static ISR and the sensitivity of insulin secretion to plasma glucose in response to the OGTT were significantly higher in participants with obesity than in normal-weight participants across all treatments (all *p* < 0.05; Table 3 and Figure A4 in Appendix B). Treatment did not significantly affect the sensitivity of insulin secretion to plasma glucose or total or static ISR responses during the OGTT in either weight group. Sucralose modified the ISR dynamic response to the glucose load only in the group with obesity (Treatment × Weight Group × Time: F_(20,360)_ = 2.19; *p* < 0.02; Figure A4 in Appendix B). Compared with water, the sucralose treatment resulted in higher dynamic ISR at time 0 but lower at 20–30 min post-glucose ingestion. The dynamic ISR for sham-fed treatment was similar to that of sucralose but different from water treatment only at 10 min post-glucose load.

### 3.4. Insulin Sensitivity and Clearance

Participants with obesity were less insulin sensitive (*p* < 0.0005) and tended to have a slower rate of insulin clearance (*p* = 0.09) than normal-weight participants (Table 3). Sucralose differentially affected S_I_ in participants with obesity and in normal-weight participants (Treatment × Weight- group: F_(2,36)_ = 4.32; *p* < 0.02). Compared with water, sucralose increased S_I_ by 52 ± 21% in normal-weight participants (*p* < 0.05). Sham-fed sucralose also increased S_I_ by 27 ± 13% in this group, but the difference from water treatment was not statistically significant (*p* = 0.18). Neither treatment significantly affected S_I_ in participants with obesity (Table 3). Ingested sucralose and sham-fed sucralose tended to increase the insulin clearance rate after ingesting the glucose load (*p* = 0.08; Table 2) and there was no significant interaction between treatment and weight group (*p* = 0.10).

### 3.5. Glucose Kinetics

**Figure 3:**
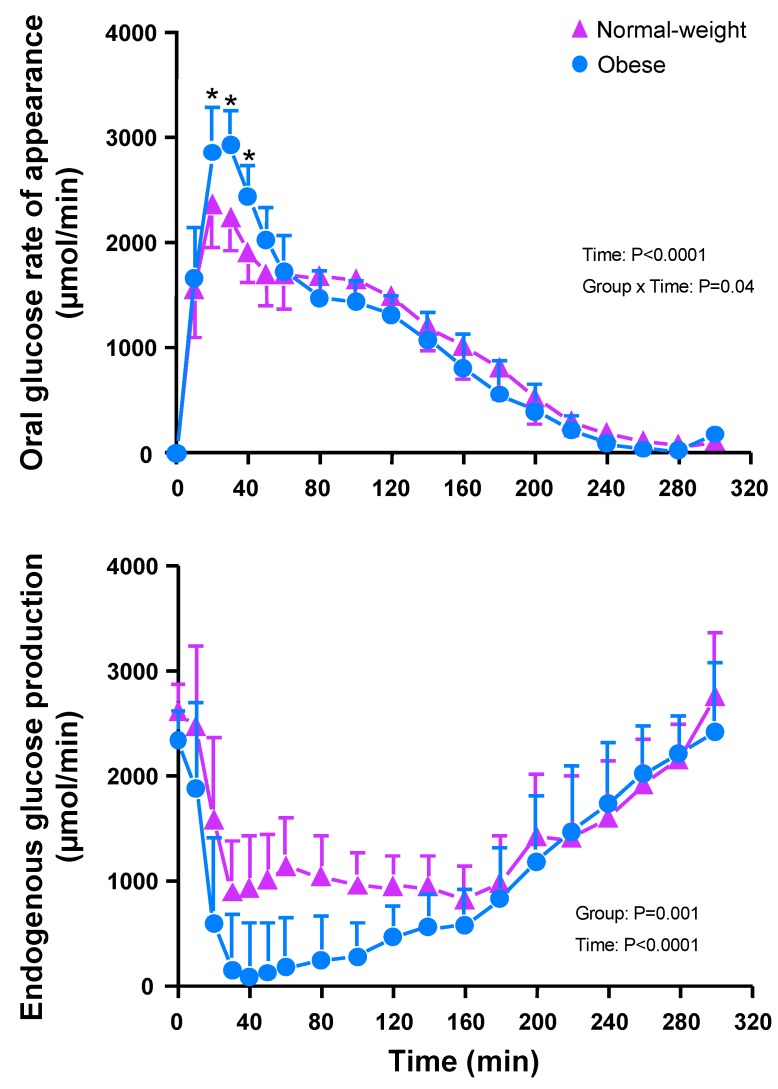
Oral glucose rate of appearance into the systemic circulation (top panel) and endogenous glucose production rate (bottom panel) after ingesting either sucralose or water or tasting but not swallowing sucralose (sham-fed) 10 min before ingesting a drink containing 75 g glucose at time 0 min. The general linear mixed model (PROC MIXED) analysis, which can handle an unbalanced designed, was used. Treatment (water, sucralose, and sham-fed), time, and weight group (normal- weight, *n* = 10, and obese, *n* = 11), as well as all interactions were included in the model and treated as fixed effects and subject was included as random effect. Post hoc Fisher least significant difference was used when values were statistically significant. * Value from group with obesity significantly different from group with normal weight, *p* < 0.05. All values are the mean ± SEM.

**Figure A5:**
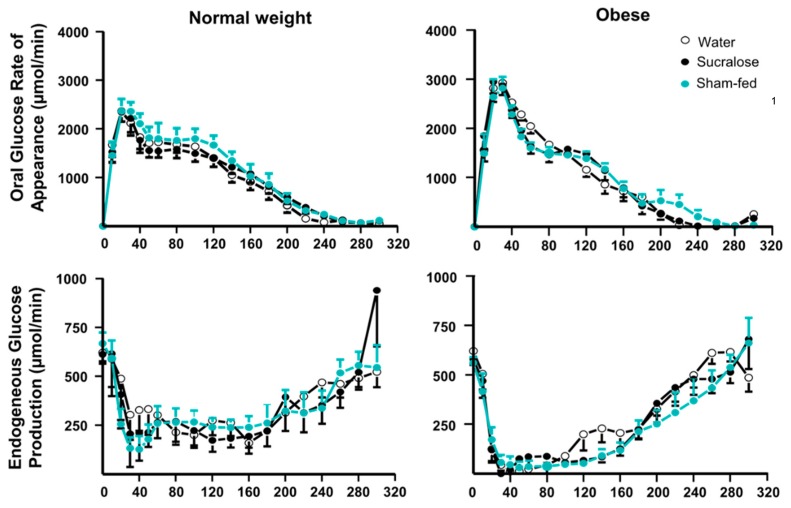
Oral glucose rate of appearance in systemic circulation and endogenous glucose production rate in normal-weight participants (*n* = 10; left panel) and participants with obesity (*n* = 11; right panel) after sucralose, water, or sham-fed treatment prior to ingestion of a 75 g glucose load at time 0 min. ^1^ *n* = 9 participants for sucralose sham-fed condition in the obese group. The general linear mixed model (PROC MIXED) analysis with subject-level random intercepts was used. Treatment (water, sucralose, and sham-fed), time, and weight group (normal-weight and obese), as well as all interactions, were included in the model and treated as fixed effects. Post hoc Fisher least significant difference was used when values were statistically significant. Data are presented as the mean ± SEM.

Participants with obesity had a higher oral glucose rate of appearance during the first 40 min of the OGTT than normal-weight participants (Weight Group × Time: F_(11,209)_ = 1.88, *p* = 0.04; Figure 3); however, the iAUC of total glucose rate of appearance and of oral glucose rate of appearance were similar between weight groups (Table 2). Glucose ingestion suppressed EGP rapidly in both groups (*p* < 0.0001), but more so in participants with obesity (*p* = 0.018; Figure 3). Sucralose, ingested or sham-fed, did not affect the total glucose rate of appearance (Figure 1) or the oral glucose rate of appearance in either weight group (Figure A5 in Appendix B). For all treatments, and in both weight groups, the amount of ingested glucose reaching systemic circulation over 5 h was ~70% of the 75 g given (Table 2).

### 3.6. Plasma Sucralose Concentrations

**Figure 4:**
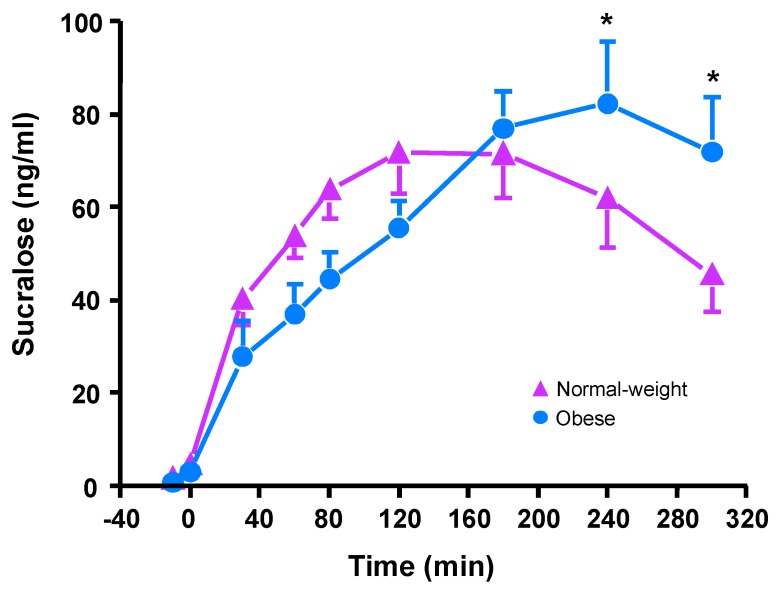
Plasma sucralose concentrations in normal-weight participants (*n* = 10) and participants with obesity (*n* = 10) after sucralose treatment prior to ingestion of a 75 g glucose load at time 0 min. The general linear mixed model (PROC MIXED) analysis with subject-level random intercepts was used. Time and weight group (normal weight and obese), as well as the interaction, were included in the model and treated as fixed effects. Post hoc Fisher least significant difference was used when values were statistically significant. * Value for participants with obesity different from normal-weight participants, *p* < 0.05. All values are the mean ± SEM.

Participants with obesity had higher plasma sucralose concentrations than did normal-weight participants during the last hour of the OGTT (Weight Group x Time: F_(9,162)_ = 2.98, *p* = 0.004; Figure 4). However, there were no significant differences between weight groups on peak sucralose concentrations (*p* = 0.31), time to peak (*p* = 0.11), or sucralose iAUC (*p* = 0.90; Table 4). We did not have data on plasma sucralose concentrations from one participant in the group with obesity and therefore only 10 participants were included in the final sample for this analysis.

## 4. Discussion

The primary finding of this study is that the ingestion of sucralose, in a quantity equivalent to that in a commercial can of soda, has different effects on postprandial glucose metabolism in participants with obesity and in normal-weight participants—none of whom regularly consume LCSs. Sucralose ingestion similarly increased glucose iAUC by ~30% in both weight groups but had opposite effects on plasma insulin concentrations. That is, in normal-weight participants, sucralose ingestion or sham-fed sucralose modestly decreased plasma insulin concentrations within the first hour after a glucose load, whereas in participants with obesity, ingested sucralose caused significantly higher insulin concentrations than sham-fed sucralose. While participants with obesity achieved higher plasma sucralose concentrations than normal-weight participants 4–5 h post-sucralose ingestion, it is unlikely that such difference could explain the divergent insulin responses between weight groups observed 1 h post-sucralose ingestion. The mechanisms responsible for the sucralose effect on glucose iAUC are unclear. We hypothesized that sucralose could affect plasma glucose excursions after the OGTT by altering endogenous glucose production (EGP), but we found that sucralose, neither ingested nor sham-fed, did not affect EGP or the rate of glucose appearance derived from ingested glucose in either weight group.

In addition, we found that the extra activation of sweet taste sensory stimulation, by sham-fed sucralose before ingesting a glucose drink, dampened plasma insulin concentrations in both groups. The mechanism underlying this sucralose sham-fed associated dampened insulin response is unknown. Also unknown is whether solutions of other isointense non-sweet taste stimuli or other sweeteners would also cause this effect. However, findings from previous studies suggest that oral sensory stimulation, by triggering a cephalic phase insulin response (CPIR), can alter plasma glucose and insulin concentrations after intragastric glucose infusions to improve glucose metabolism in healthy lean men [30]. Further, brief intravenous infusion of insulin in a pattern that mimics a CPIR during food ingestion improves glucose tolerance in people with obesity [31]. Although sucralose did not trigger a measurable CPIR during the first 10 min post ingestion (neither in this study nor in our previous study [18]), a possibility that we did not evaluate is that tasting intense sweetness 10 min before an OGTT sensitized a CPIR, or other cephalic hormonal responses, to the ingestion of glucose. Interestingly, recent findings from clinical studies that used neuroimaging and indirect calorimetry also suggest that oral perception of sweetness plays a pivotal role in the regulation of carbohydrate metabolism [32]. Through a series of elegant studies, Veldhuizen et al. [32] manipulated sweetness levels independently from caloric load to demonstrate that the optimal metabolic response to a carbohydrate load (and its rewarding value) depends on the perfect match between the intensity of its sweetness and its energy content. Finally, our finding that sweetness perception before a glucose load decreased insulin response after an OGTT, complements findings from pre-clinical and clinical studies that show the opposite insulin response when subjects are deprived of sweet taste perception [33,34]. That is, the inhibition of sweet taste perception, either by direct administration of glucose to the stomach (i.e., bypassing oral taste perception) in rats [33], or by adding lactisole (a broad antagonist of the sweet taste receptor) to a glucose load in normal-weight participants [34] heightened insulin responses to an OGTT. However, it should be noticed that, as in this last study [34], lactisole was added to the oral glucose load, and it is unclear whether inhibition of sweetness signaling in the mouth and/or the gut altered postprandial insulin concentrations. For example, findings from a study in healthy lean subjects who received intragastric infusions of glucose with and without lactisole suggest that sweet taste receptors expressed in the gut can affect glucose metabolism, independently of sweetness signaling in the mouth [35]. In this study, lactisole, despite attenuating glucose-stimulated glucagon-like peptide-1 secretion, increased glucose AUC without changing insulin AUC, which suggest that post-oral sweetness signaling can affect insulin sensitivity [35]. Although more studies are needed to disentangle the effects of activating oral versus intestinal sweet taste signaling in glucose metabolism, collectively, these findings offer an innovative mechanism by which LCSs might upset metabolic fate of carbohydrates. We hypothesize that overly sweetened diets alter sweet taste signaling systems that play a role in the regulation of postprandial glucose metabolism, helping explain, at least in part, why high consumption of LCSs is associated with the same detrimental health effects as high consumption of added sugars, including an increased risk of developing type 2 diabetes [4].

Compared with water treatment, sucralose ingestion increased glucose-stimulated insulin responses only in participants with obesity. However, the increase in plasma insulin concentrations was of a smaller magnitude than in our previous study [18] (which may also explain why plasma glucose concentrations did not reach as low of a nadir). We hypothesize that differences in the magnitude of the insulin response may be related to the inclusion of a new treatment condition in this current study design (i.e., sucralose sham-fed), which had a significant (and opposite) effect in both weight groups. Inconsistent with our previous findings that sucralose ingestion before an OGTT significantly reduced S_I_ (by 23 ± 20%) in participants with obesity, here, we found that sucralose reduced S_I_ in this weight group by only 12 ± 8%, which was not statistically significant. Remarkably, acute sucralose ingestion significantly increased S_I_ by 52 ± 20% in normal-weight participants. The mechanism responsible for this acute effect of sucralose is unknown, but our data suggest that it is not likely to be explained by changes in hepatic insulin sensitivity. Neither ingested nor sham-fed sucralose affected the rate of EGP in either weight group. However, contrary to our finding of an increased S_I_ after acute consumption of sucralose in normal-weight participants, two independent groups recently reported that chronic consumption of sucralose (i.e., daily for 2 weeks) in such a group decreased S_I_ [36,37]. Importantly, all these studies (including our own) included participants who were non-habitual consumers of LCSs at baseline. It is therefore plausible to hypothesize that acute effects of sucralose on S_I_ are different from chronic effects due to the development of compensatory responses (i.e., tolerance) to the consumption of sucralose. Typically, once tolerance to a substance has been developed, exposure to a smaller dose of such a substance, or to stimuli conditioned to the presence of the substance (such as its “taste”) triggers the compensatory response, which is commonly opposite to the initial acute effects of the substance [38].

Sucralose did not significantly affect total glucose rate of appearance or oral glucose rate of appearance in either weight group. Interestingly, although total glucose rate of appearance was similar between groups, in participants with obesity the oral glucose rate of appearance was increased, and the rate of EGP was decreased during the first 40 min of the OGTT. The finding of a higher oral glucose rate of appearance in participants with obesity is consistent with results of previous studies that demonstrated increased expression of the sodium glucose transporter isoform 1 (SGLT1) in the duodenum of people with severe obesity [39]. Such an increased expression of SGLT1 has been associated with a faster rate of intestinal glucose absorption [39,40] and is likely to account, at least partially, for the observed insulin hypersecretion. In turn, the observed hyperinsulinemia after consuming the glucose drink in the group with obesity might explain the remarkable difference in EGP between groups, because EGP is very sensitive to small increases in circulating insulin [41]. That the group with obesity, which is more insulin resistant, suppressed EGP more than the group with normal weight, which is more insulin sensitive, may sound paradoxical. However, data suggest that, in participants without diabetes, EGP decreases (not increases) with increases in Body Mass Index) [42].

An important strength of this study design is also a limitation: our entire study population comprised people who do not normally consume LCSs. The logic for this inclusion criteria was based on the finding from several preclinical studies that chronic LCS ingestion affects intestinal glucose uptake [5,6,7] and glycemic responses to an oral glucose load [43,44]. In addition, following from our previous findings [18], the group with obesity comprised people with HOMA-IR2 < 3. However, our findings might not extrapolate to people who habitually consume LCSs. Additional studies with regular LCS consumers, people who are more insulin resistant, and people with diabetes are needed. It should also be noticed that menstrual stage is known to affect incretin axis physiology and we did not control for menstrual stage in our female participants. Finally, for all treatments, except for the sham-feeding procedure, participants ingested 60 mL of fluid 10 min before the OGTT. Therefore, it may be that the difference of 60 mL of fluid preload between treatments could have altered (a) gastric emptying or (b) the concentration of ingested glucose in the intestine during the glucose challenge. However, we believe this to be unlikely because data from several studies demonstrate that gastric emptying of glucose is generally independent of the volume and concentration of the glucose solution [45,46,47] and is tightly regulated by post-pyloric signals that result in glucose entering the duodenum at an approximately constant rate [45].

## 5. Conclusions

The results from our study, while very modest in magnitude, add to a growing body of literature demonstrating that sucralose is not metabolically inert and contribute further to the observation that sucralose differentially affects glucose metabolism in people with obesity and people with normal weight. In addition, our findings underscore a physiological role for taste perception in postprandial glucose responses, which supports the notion that sweeteners, regardless of their associated caloric contribution, should be consumed in moderation.

## Figures and Tables

**Table 1:** Participant characteristics.

Characteristic	Normal Weight (*n* = 10)	Obese (*n* = 11)
Age (years)	27.0 (4.2)	29.5 (4.0)
Weight (kg)	66.0 (10.7)	102.9 (15.3) *
BMI (kg/m^2^)	22.8 (0.9)	37.7 (5.5) *
Female/male	7/3	10/1
Race		
White	7 (70%)	5 (45%)
Black	2 (20%)	5 (45%)
Other	1 (10%)	1 (10%)
Plasma concentrations		
Fasting glucose (mmol/L)	5.0 (0.3)	5.1 (0.3)
Fasting insulin (pmol/L)	38.1 (15.8)	87.0 (35.6) *
HOMA-IR2	0.7 (0.1)	1.7 (0.2) *
Prediabetes ^1^	3 (30%)	5 (45%)

Data are the mean (SD), except race and prediabetes data, which are n (%). An unpaired t-test was used to compare baseline characteristics between weight groups. * Significant difference from normal-weight group (*p* < 0.05). ^1^ Prediabetes was defined as impaired fasting glucose and/or impaired glucose tolerance on the day the oral glucose tolerance test was preceded by consumption of water. HOMA-IR2: homeostatic model assessment of insulin resistance score. Body Mass Index (BMI) is the weight of a person (in kg) divided by his or her height in meters squared.

**Table 2:** Metabolic response to an oral 75 g glucose load preceded by sucralose or water ingestion or by tasting and expectorating sucralose (sham-fed) and glucose kinetics.

Measure	Normal Weight			Obese			*p* Value
	Water (*n* = 10)	Sucralose (*n* = 10)	Sham-Fed (*n* = 10)	Water (*n* = 11)	Sucralose (*n* = 11)	Sham-Fed (*n* = 9) ^1^	Group X Treatment
**Metabolic Response**							
**Incremental Peak Response**							
Glucose (mmol/L)	5.7 ± 0.4	5.4 ± 0.5	5.5 ± 0.4	4.4 ± 0.4	4.7 ± 0.4	4.5 ± 0.4	0.27
Insulin (pmol/L) *	683 ± 130	548 ± 91	590 ± 115	1062 ± 124	1193 ± 171	1041 ± 184	0.12
C-peptide (nmol/L) *	2.8 ± 0.3	2.6 ± 0.3	2.8 ± 0.3	3.7 ± 0.4	3.9 ± 0.4	3.6 ± 0.3	0.20
GIP (pmol/L)	49.5 ± 6.8	45.0 ± 5.9	48.5 ± 5.5	61.9 ± 4.0	59.6 ± 5.2	56.0 ± 4.4	0.53
**iAUC**							
Glucose (mmol/L)·300 min ^†^	470 ± 56	560 ± 66	491 ± 60	380 ± 55	455 ± 48	455 ± 59	0.81
Insulin (10^2^ pmol/L)·300 min *	654 ± 131	607 ± 102	597 ± 106	1195 ± 177	1400 ± 241	1282 ± 257	0.10
C-peptide (nmol/L)·300 min *	375 ± 40	394 ± 43	375 ± 45	502 ± 54	565 ± 47	541 ± 43	0.37
GIP (10^2^ pmol/L)·300 min	67 ± 11	62 ± 10	64± 7	79 ± 6	74 ± 5	72 ± 7	0.91
**Glucose Kinetics**							
**Peak Response**							
Total Ra (µmol/min)	2274 ± 147	2313 ± 242	2071 ± 253	2576 ± 206	2672 ± 198	2552 ± 229	0.88
Oral Ra (µmol/min) *	2565 ± 109	2436 ± 154	2482 ± 203	3159 ± 217	3169 ± 225	3094 ± 244	0.67
**AUC**							
Total Ra (10^2^ mol/min)·300 min	3947 ± 269	3920 ± 296	4348 ± 315	3664 ± 85	3580 ± 91	3436 ± 95	0.10
Oral Ra(10^2^ mol/min)·300 min	2884 ± 143	2967 ± 69	3405 ± 185	2910 ± 90	2928 ± 98	2780 ± 87	0.16
%Total oral glucose appearing in circulation (out of 75 g)	69.6 ± 3.5	71.3 ± 1.7	78.9 ± 4.7	69.7 ± 2.5	71.2 ± 2.8	71.3 ± 3.1	0.21

Data are the mean ± SEM. Data were analyzed using the general linear mixed model (PROC MIXED) with sub-level random intercepts. Treatment (water, sucralose, and sham-fed), and weight group (normal weight and obese), as well as the interaction were included in the model and treated as fixed effects. ^1^ PROC MIXED analysis can handle unbalanced designs so data from the two participants who did not complete one of the study visits were also included. * Significant main effect of group, *p* < 0.05; ^†^ Significant main effect of treatment: sucralose different from water, *p* < 0.05. GIP: plasma glucose-dependent insulinotropic peptide; Total Ra: total glucose rate of appearance; Oral Ra: oral glucose rate of appearance.

**Table 3:** β-Cell Response, insulin sensitivity and clearance in participants with normal weight and participants with obesity.

Measure	Normal Weight			Obese			*p* Value
	Water (*n* = 10)	Sucralose (*n* = 10)	Sham-Fed (*n* = 10)	Water (*n* = 11)	Sucralose (*n* = 11)	Sham-Fed Water (*n* = 9) ^1^	Group X Treatment
β-Cell Function							
Φ_total_ (10^9^·min^−1^) *	13.9 ± 0.8	13.6 ± 1.1	14.0 ± 1.0	23.3 ± 1.9	23.9 ± 1.9	24.1 ± 2.0	0.74
Φ_dynamic_ (10^9^) *	513 ± 114	571±117	483 ± 75	1269 ± 201	936 ±148	977 ± 200	0.12
Φ_static_ (10^9^·min^−1^) *	45.6 ± 8.3	43.3 ± 5.4	40.7 ± 5.9	70.2 ± 12.7	70.8 ± 10.4	63.4 ± 7.2	0.58
Insulin Sensitivity *							
S_I_ [10^−5^ dl·kg^−1^·min^−1^/(pmol/L)]	16.3 ± 3.0 ^a^	25.5 ± 6.1 ^b^	19.7 ± 2.9 ^a^	5.6 ± 0.8 ^c^	4.8 ± 0.7 ^c^	5.6 ± 0.5 ^c^	0.02
Insulin Clearance							
Clearance rate (L·min^−1^)	1.7 ± 0.2	1.9 ± 0.2	1.9 ± 0.2	1.5 ± 0.1	1.5 ± 0.1	1.5 ± 0.1	0.10

Data are the mean ± SEM. Data were analyzed using the general linear mixed model (PROC MIXED) with sub-level random intercepts. Treatment (water, sucralose, and sham-fed), and weight group (normal weight and obese), as well as the interaction were included in the model and treated as fixed effects. ^1^ PROC MIXED analysis can handle unbalanced designs so data from the two participants who did not complete one of the study visits were also included. * Significant main effect of group, *p* < 0.05; Different letters indicate significant difference between the treatments, *p* < 0.05 (post hoc Fisher least significant difference after interaction effect Group × Treatment, *p* < 0.02).

**Table 4:** Plasma sucralose kinetics in participants with normal weight and participants with obesity.

Sucralose Kinetics	Normal-Weight	Obese
Peak Response (ng/mL)	78.6 ± 8.4	93.2 ± 11.1
Time to Peak (min)	143 ± 19	194 ± 24
AUC (10^2^ ng/L·300 min)	175 ± 19	176 ± 145

## Appendix A

### Supplementary Methods

Glucose kinetics. Total glucose rate of appearance and endogenous glucose production (EGP) were determined as previously reported [28]. In brief, during the last 20 min of the basal tracer equilibration period, EGP was calculated as the ratio of [6,6-2H2] glucose infusion rate to the plasma tracer enrichment (tracer-to-tracee ratio, TTR6,6).

After glucose ingestion, the total glucose rate of appearance was calculated from TTR6,6 using Steele’s equation for non-steady-state conditions [29], as follows:

Total Ra(t) = [(IR − C(t)) × V × (dTTR6,6(t)/dt)]/TTR6,6(t), (A1)

where IR is the infusion rate of [6,6-2H2] glucose, C is the plasma glucose concentration, and V is the volume of distribution (162.5 mL∙kg^−1^).

The plasma glucose concentration resulting from endogenous glucose release (Cend) was obtained as the difference between total and exogenous glucose concentration. TTRend of endogenous glucose was calculated as follows:

TTRend = dTTR6,6(t) × C(t)/Cend (A2)

EGP was calculated from TTRend using Steele’s equation for non-steady-state conditions:

EGP(t) = [(IR − C(t)) × V × (dTTRend (t)/dt)]/TTRend (t) (A3)

The oral glucose rate of appearance was calculated as the difference between Total Ra and EGP [28].
